# Supplementary material for: Phenotypic profiling with a living biobank of primary rhabdomyosarcoma unravels disease heterogeneity and AKT sensitivity
Source: Nat Commun. 2020 Sep 15;11:4629. doi: 10.1038/s41467-020-18388-7 (PMC7492191; doi:10.1038/s41467-020-18388-7)
Supplement: Supplementary file 1 — Supplementary Information [file 41467_2020_18388_MOESM1_ESM.pdf]

<u>**Supplementary data**</u>

Phenotypic profiling with a living biobank of primary rhabdomyosarcoma unravels disease heterogeneity and AKT sensitivity

Manzella et al.

Supplementary Figures

A

PDX ID	Histology (translocation)	Previous treatment	PPC lines	Origin
SJRHB13758_X1	ERMS	no	yes	St. Jude Children's Research Hospital, Memphis (USA)
SJRHB13758_X2	ERMS	yes	yes	St. Jude Children's Research Hospital, Memphis (USA)
SJRHB012_X	ERMS	no	no	St. Jude Children's Research Hospital, Memphis (USA)
SJRHB012_Y	ERMS	yes	yes	St. Jude Children's Research Hospital, Memphis (USA)
SJRHB012_Z	ERMS	yes	yes	St. Jude Children's Research Hospital, Memphis (USA)
SJRHB013_X	ERMS	no	yes	St. Jude Children's Research Hospital, Memphis (USA)
SJRHB011_X	ERMS	no	yes	St. Jude Children's Research Hospital, Memphis (USA)
SJRHB011_Y	ERMS	yes	yes	St. Jude Children's Research Hospital, Memphis (USA)
RMS-ZH004_X	ERMS	yes	yes	University Children's Hospital of Zurich (Switzerland)
RMS-ZH005_X	ERMS	yes	yes	University Children's Hospital of Zurich (Switzerland)
N870_X	ERMS	yes	yes	Emma Children's Hospital, Amsterdam (Netherlands)
RMS-AMS007_X	ARMS (no translocation)	yes	yes	Emma Children's Hospital, Amsterdam (Netherlands)
RMS-ZH002_X	ARMS (PAX3-FOXO1)	yes	yes	University Children's Hospital of Zurich (Switzerland)
RMS-ZH003_X	ARMS (PAX3-FOXO1)	yes	yes	University Children's Hospital of Zurich (Switzerland)
IC-pPDX-29 (MAP-IC-A64-RMS-1)	ARMS (PAX3-FOXO1)	yes	yes	Institut Curie Centre de Recherche, Paris (France)
IC-pPDX-35 (MAP-IC-A76-RMS-1)	ARMS (PAX3-FOXO1)	yes	yes	Institut Curie Centre de Recherche, Paris (France)
SJRHB010463_X16	ARMS (PAX3-FOXO1)	yes	yes	St. Jude Children's Research Hospital, Memphis (USA)
SJRHB013759_X1	ARMS (PAX3-FOXO1)	yes	yes	St. Jude Children's Research Hospital, Memphis (USA)
SJRHB013757_G1	ARMS (PAX7-FOXO1)	yes	yes	St. Jude Children's Research Hospital, Memphis (USA)
SJRHB010468_X1	ARMS (PAX7-FOXO1)	yes	yes	St. Jude Children's Research Hospital, Memphis (USA)

B

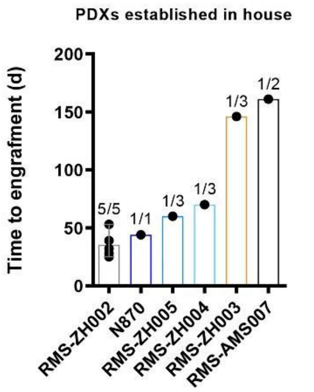

C

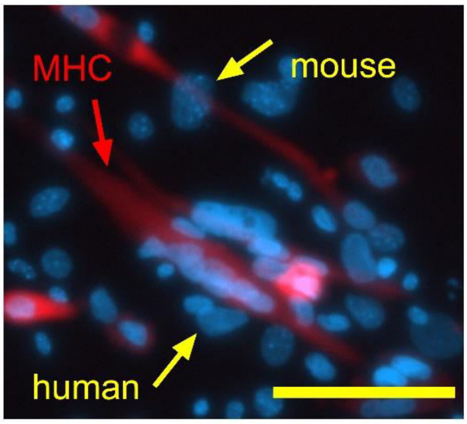

D

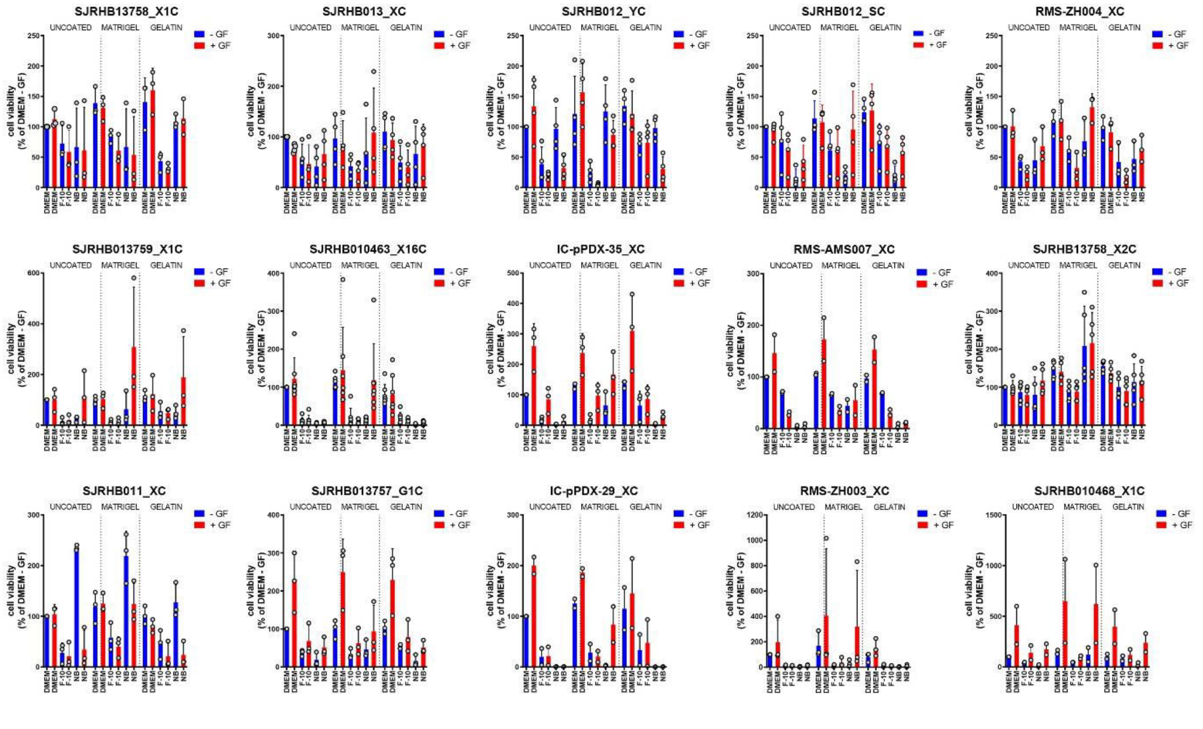

Supplementary Figure 1. Sample information and culture condition screen

(A) Description of the PDX material used in the present study including subtype, previous treatment and origin of patient/PDX material.

(B) Rate of PDX engraftment of samples established in Zurich. Graph bars indicating the day of engraftment (tumor volume ~100 mm^3) for each PDX established in house. The number of successful engraftments and the total number of mice transplanted with primary patient materials are displayed on the top of each bar (Mean ± range; n=1-5 transplanted mice).

(C) Detection of mouse and differentiated human RMS cells by immunofluorescence. Representative image of SJRHB010463_X16C cells grown in DMEM plus GF on matrigel-coated plates and stained for MHC. DAPI was used for staining of the nuclei. Mouse cells are identified according to their punctate DAPI staining pattern. MHC is used as marker for differentiation of RMS cells along the myogenic lineage. Scale bar 100 μm. The displayed image is representative of n=29 independent experiments summarized in Fig.1B.

(D) Histograms showing cell viability results presented in Figure 1B as heatmap for individual data points. Blue and red graph bars indicate absence or presence of supplemented growth factors (GF, bFGF plus EGF), respectively. (RMS-AMS007, IC-pPDX-29 and SJRHB010468_X1, mean ± range, n=2 biological replicates; SJRHB13758_X1, RMSZH004, IC-pPDX-35, SJRHB011_X, SJRHB013757_X2 and RMS-ZH003, mean ± sd, n=3 biological replicates; SJRHB013_X, SJRHB012_Y, SJRHB012_Z and SJRHB13758_X2, mean ± sd, n=4 biological replicates; SJRHB010463_X16, mean ± sd, N=6 biological replicates).

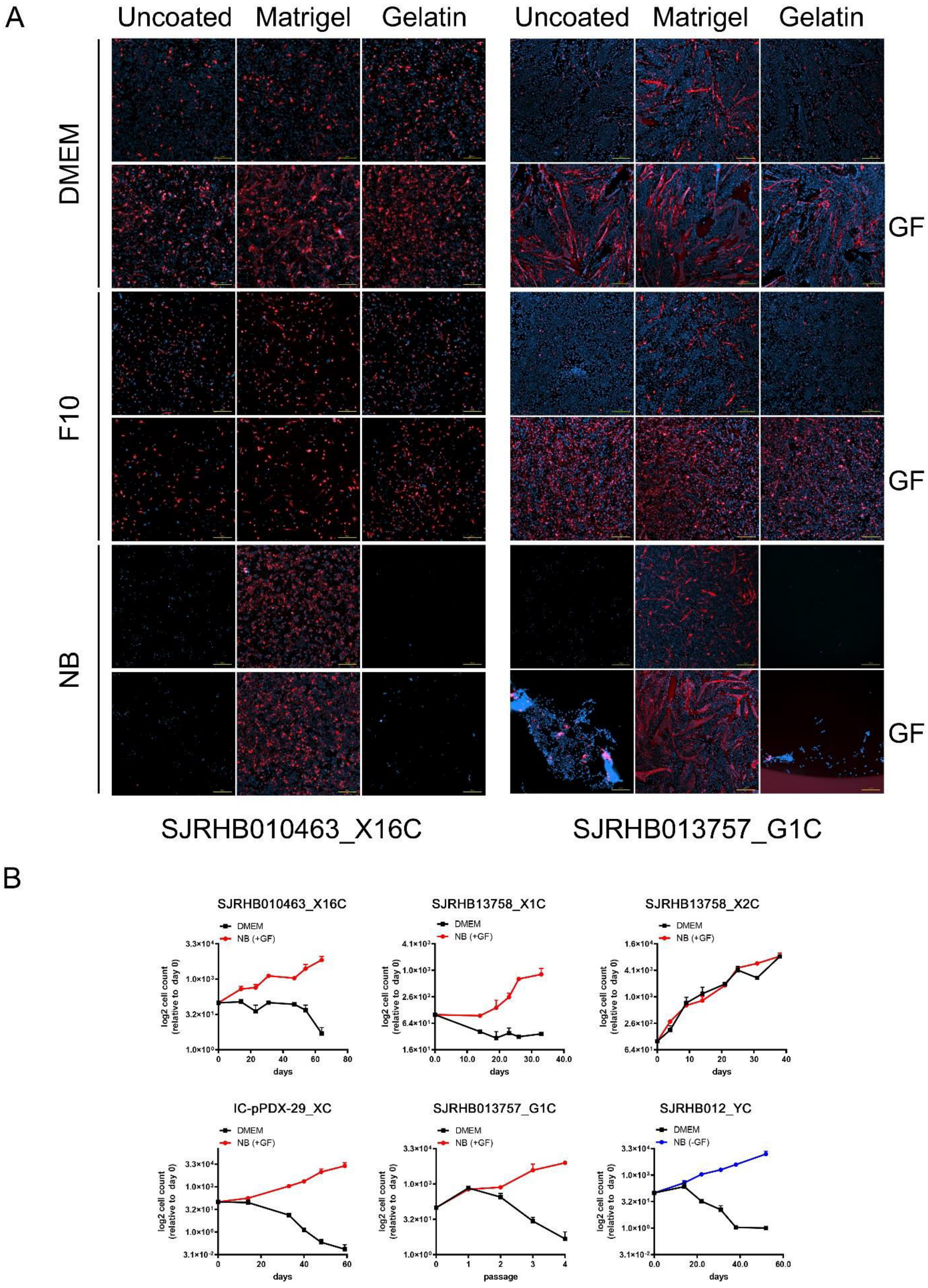

Supplementary Figure 2. Terminal skeletal muscle differentiation assessment and long-term proliferation analysis of PPCs

(A) IF analysis of SJRHB010463_X16C (left panel) and SJRHB013757_G1C (right panel) cells stained for MHC (red) under indicated culture conditions. DAPI was used to stain the nuclei (blue). Scale bars 200 μm. The displayed pictures are representative of n=2 biological replicates.
(B) Cell numbers measured by counting over time of cells cultured under indicated conditions. DMEM (black line), NB-GF (blue line), NB+GF (red line). Data are expressed in a logarithmic scale (log2). (Mean $\pm$ range; n=2 biological replicates).
Source data are provided as source data file.

A

IC-pPDX-35_XC

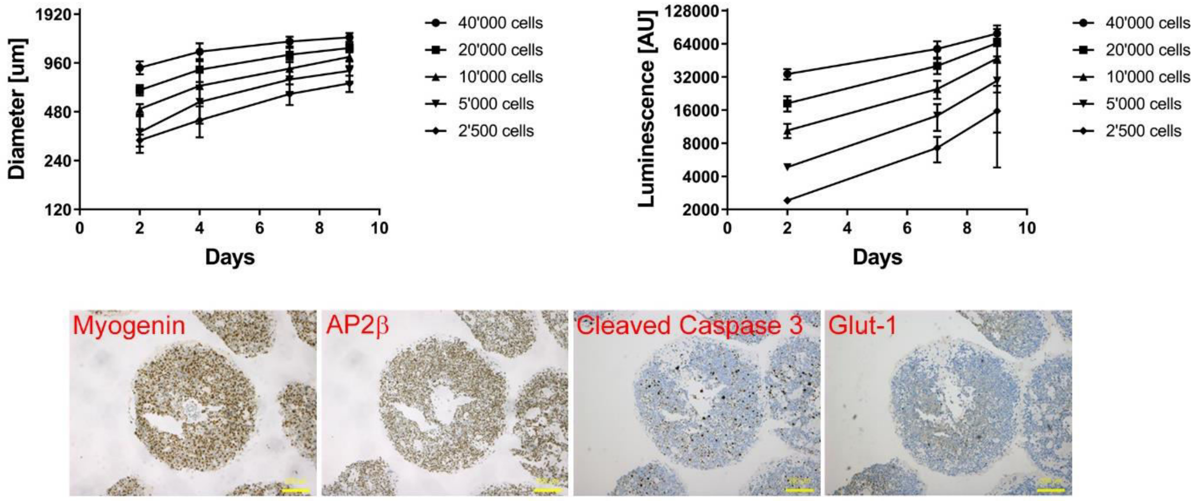

B

SJRHB13758_X2C

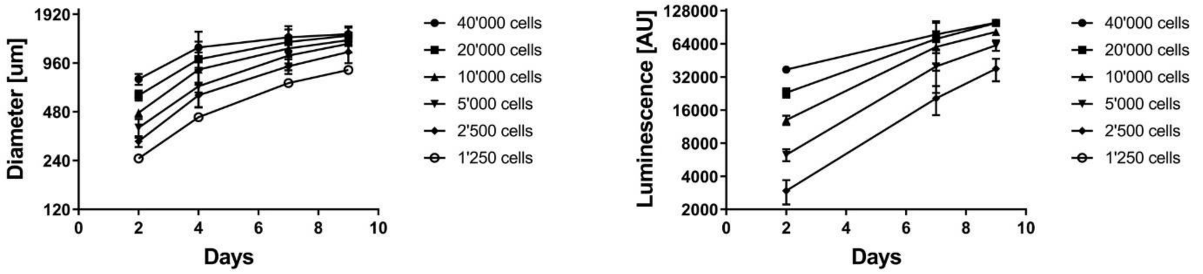

<u>Supplementary Figure 3</u>. Growth of PPCs as spheroids

(A) Growth and morphology of IC-pPDX-35 spheroids. Upper left and right panel, Increase of spheroid diameter and cell content as determined by an ATP dependent cell viability assay over a period of 9 days is depicted. Number of cells plated at day 0 are indicated on the right. (Mean±sd; n=3 biological replicates). Lower panel, Expression of markers for RMS (Myogenin and AP2β), apoptosis (cleaved Caspase-3) and hypoxia (Glut-1) was determined by immunohistochemistry using 7 day old spheroids. (B) Growth of SJRHB13758_X2C spheroids. Number of cells plated at day 0 is indicated on the right. Increase of spheroid diameter (left panel) and cell content as determined by an ATP dependent cell viability assay (right panel) over a period of 9 days is shown. (Mean ± sd; n=3 biological replicates). Source data are provided as source data file.

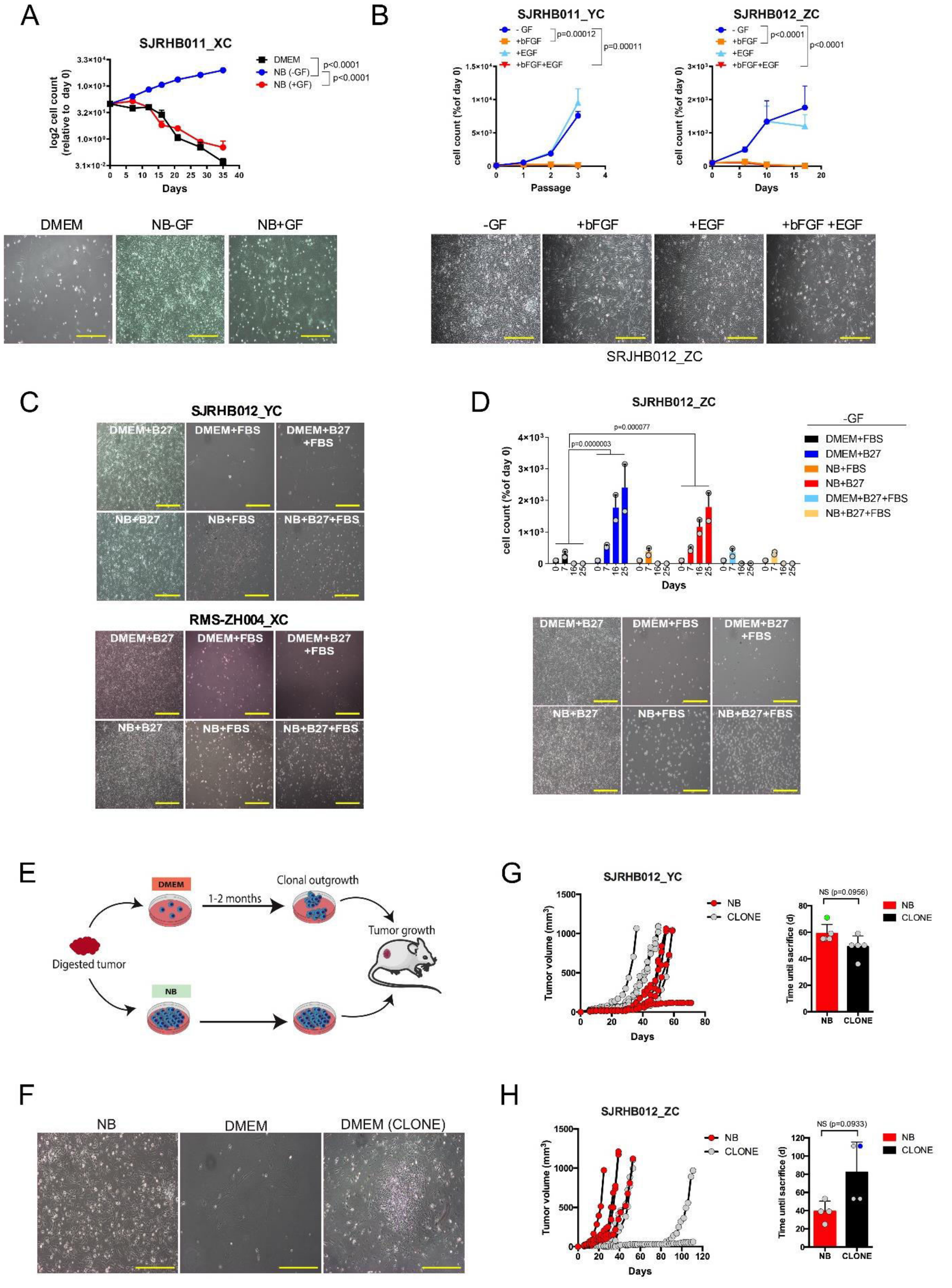

Supplementary Figure 4. Effect of growth factors and serum on additional PPCs

(A, upper panel) Number of cells relative to day of PDX dissociation (day 0) for SJRHB011_XC under described culture conditions and on matrigel-coated plates. (Mean ± sd; n=3 biological replicates; two-way ANOVA with Tukey`s multiple comparisons test). (A, lower panel) Representative images showing

SJRHB011_XC cells at passage 3 cultured under standard conditions (DMEM), NB-GF and NB+GF. Scale bars 200 μm

(B, upper panel) Growth of SJRHB011_YC and SJRHB012_ZC cells cultured under indicated conditions. SJRHB011_YC, mean ± range; n=2 biological replicates and SJRHB012_ZC, mean ± sd , n=3 biological replicates; two-way ANOVA with Tukey`s multiple comparisons test). (B, lower panel) Representative photographs from cells at passage 1 cultured under indicated conditions. Scale bars 200 μm.

(C) Representative bright-field images taken at passage 3 from experiments in shown Figure 2D. Scale bars 200 μm.

(D, upper panel) Cell counts of SJRHB012_ZC cells cultured under indicated conditions. -GF indicate the absence of supplemented growth factors. (Mean ± range; n=2 biological replicates; Sidak`s multiple comparisons test). (D, lower panel) Photographs of SJRHB012_ZC cells at passage 3. Scale bars 200 μm.

(E) Scheme depicting the difference in selection pressure imposed by DMEM and NB conditions. Serum induced toxicity leads to death of most cultured cells and outgrowth of clones upon a long-time period (1-2 months). On the contrary, NB-derived cells are not associated with such selection.

(F) Example of the phenomenon depicted in (E). Images were taken from SJRHB012_ZC cells grown in NB and DMEM (100X). DMEM tolerant clones (CLONE) appeared after 1-2 months in culture. Scale bars 200 μm. Images are representative of n=2 independent experiments.

(G and H, left panel) Tumor growth kinetics of indicated xenografts derived from matched NB cells (red) and DMEM_clones (gray) (SJRHB012_Y, n=5 xenografts; SJRHB012_Z, n=4 xenografts). (G and H, right panel) Graph bars indicating day (d) of sacrifice for mice from (G and H, left panel). Gray dots indicate the day of sacrifice for individual mice when tumors reached ~1000mm^3. Blue and green dots depict the time of sacrifice for mice with no tumors or with tumors that did not reach the endpoint within the timeframe of the experiment, respectively. (Mean ± sd; SJRHB012_Y, n=5 xenografts; SJRHB012_Z, n=4 xenografts; two-tailed paired t test; NS, not significant).

Source data are provided as source data file.

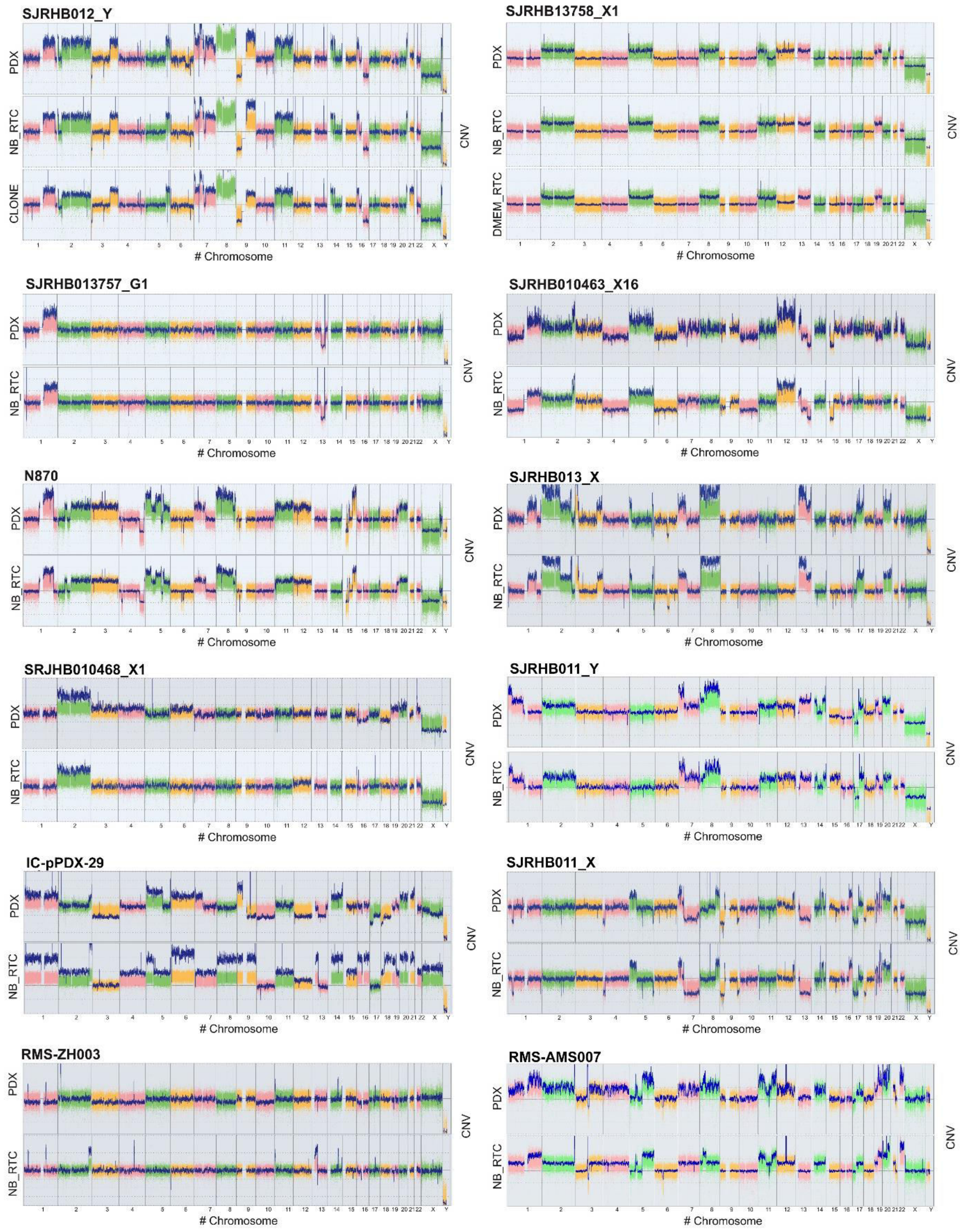

<u>Supplementary Figure 5</u>. aCGH analysis of PDX and corresponding PPCs

Copy-number variant (CNV) analysis of PDXs and corresponding PPCs (passage <11).

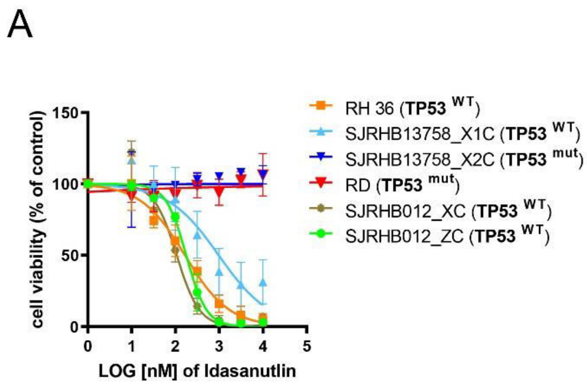

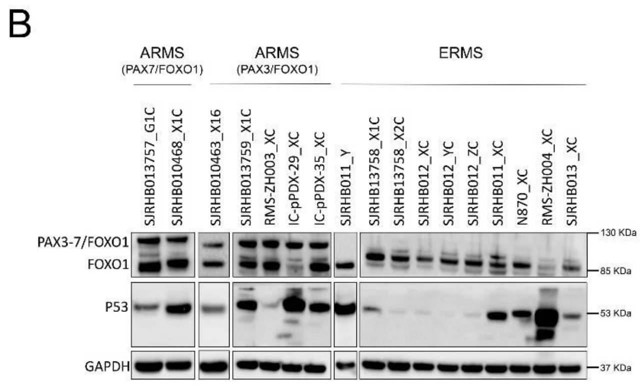

<u>Supplementary Figure 6</u>. Molecular analysis of PDX and corresponding PPCs

(A) Cell viability analysis of TP53 wild type (TP53^{wt}) and TP53 mutated (TP53^{mut}) cells treated with increasing concentrations of Idasanutlin as measured by WST-1 assay (Rh36, SJRHB13758_X1C, SJRHB13758_X2C, RD, Mean ± sd, n=3 biological replicates; SJRHB012_XC, SJRHB012_ZC, mean± range, n=2 biological replicates).

(B) Western blot analysis of cell extracts from 17 different PPCs. PAX3/FOXO1 and P53 protein levels are shown. GAPDH was used as loading control. Displayed blots are representative of n=2 independent detections.

Source data are provided as source data file.

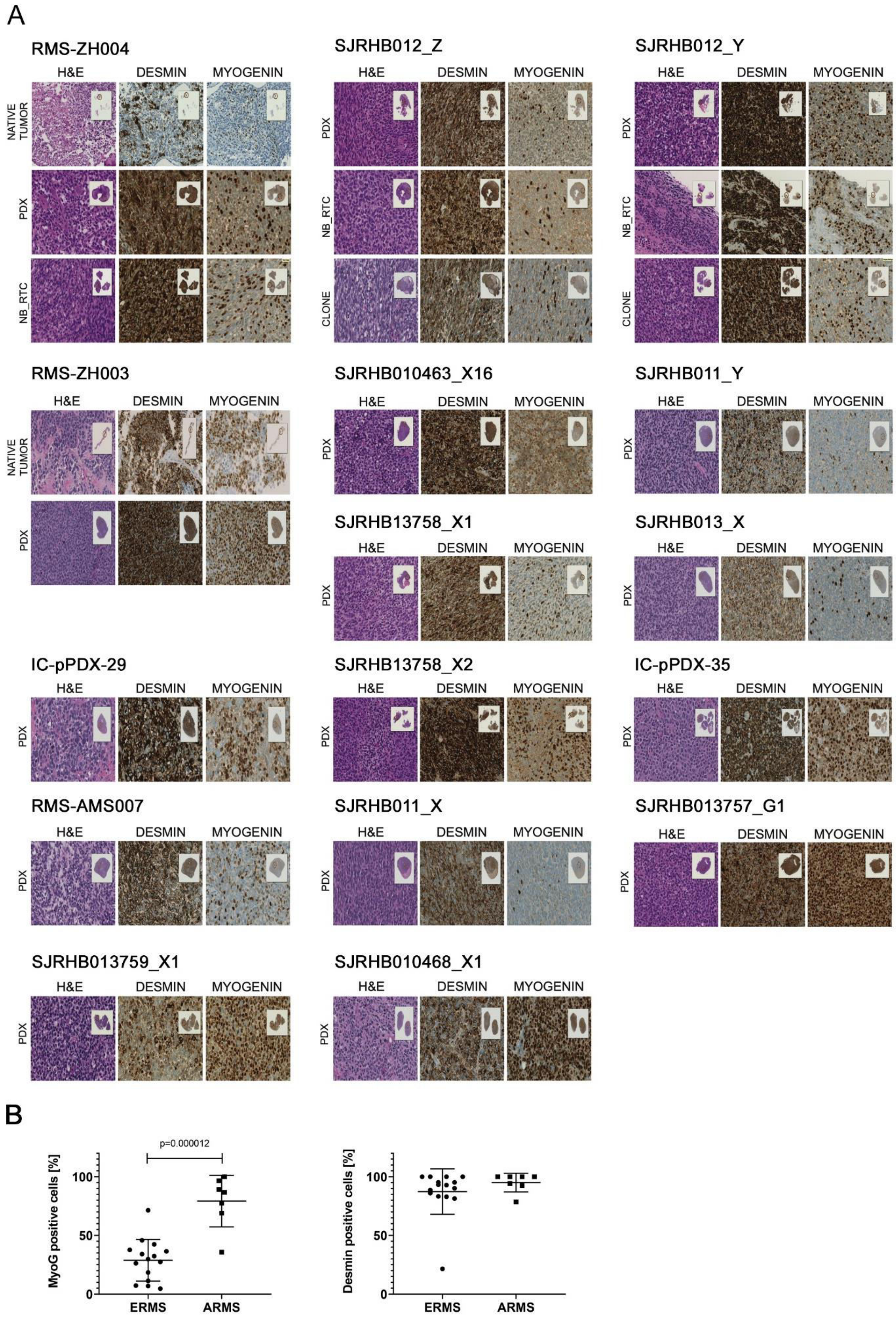

Supplementary Figure 7. Histology of PDXs and PPC-derived xenografts

(A) Images of sections from FFPE tumor xenografts stained for Hematoxylin and Eosin (H&E), DESMIN and MYOGENIN. 200X magnification. Displayed images are representative of n=1 stainings. (B) Quantification of MYOGENIN and DESMIN positive cells in stainings shown in (A). (Mean ± sd; ERMS, n=15 tumors; ARMS, n=7 tumors; Two-tailed unpaired t test).

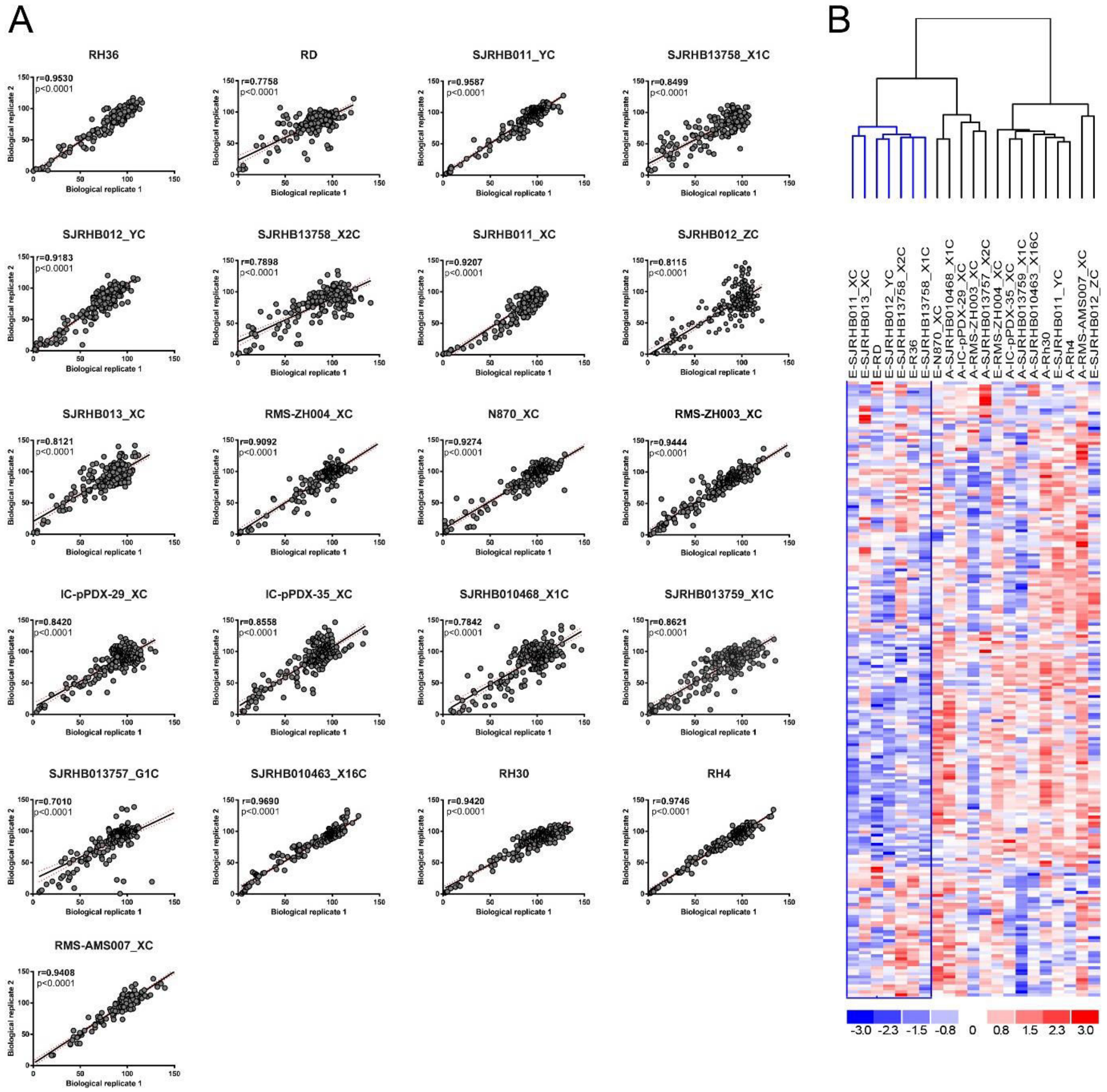

<u>Supplementary Figure 8</u>. Drug response data correlations and unsupervised hierarchical clustering

(A) Pearson correlation coefficient (r) between two biological replicates for each agent used in the drug screen (shown in Figure 4A) by considering the normalized cell viability values relative to DMSO control following drug treatment (the p value is shown in each graph).

(B) Unsupervised hierarchical clustering analysis performed with the drug response data shown in Figure 4A. The activity of the compounds (row) is shown for each sample (column).

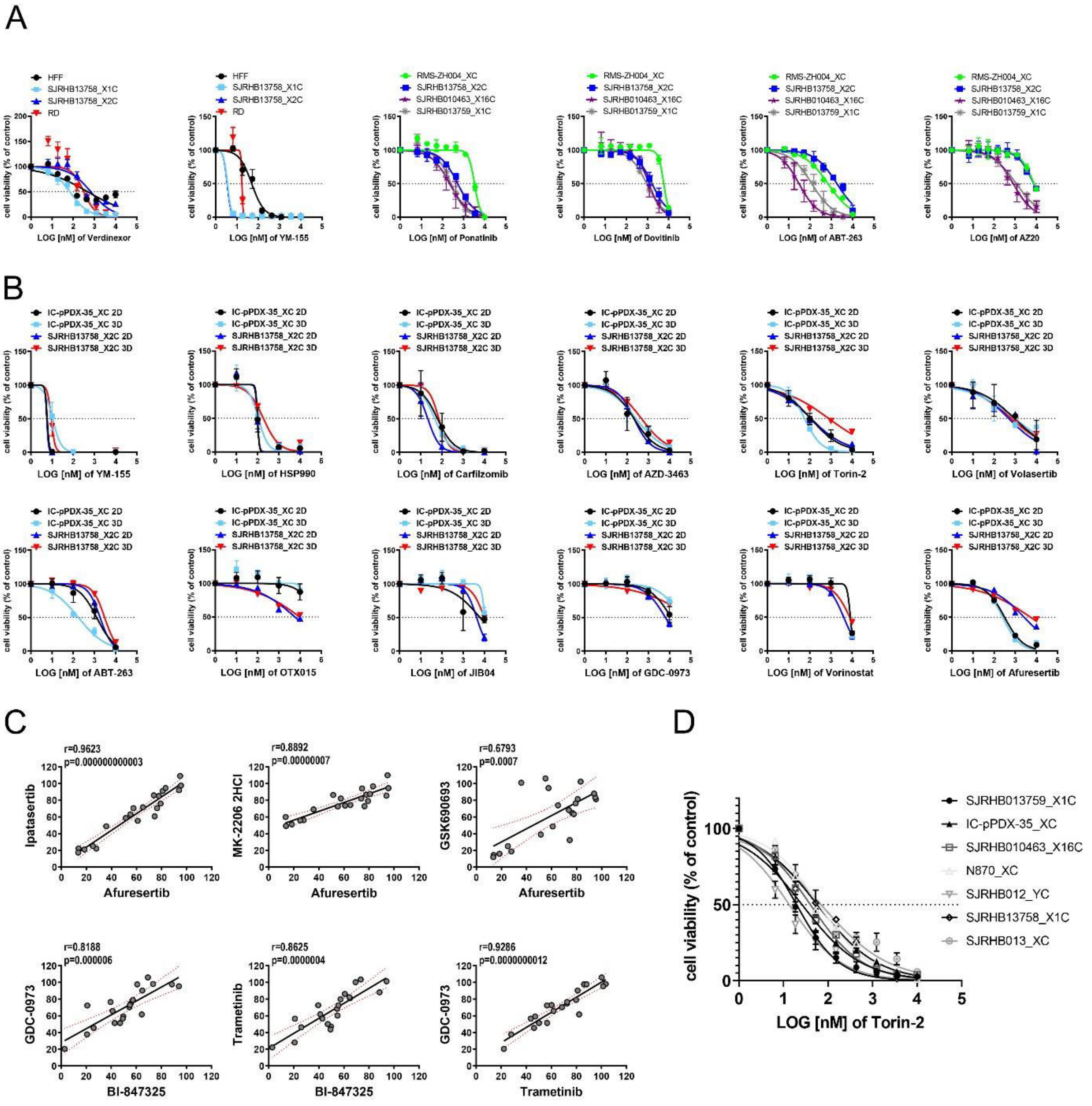

Supplementary Figure 9. Drug response curves for selected drugs applied in 2D and 3D cultures and analysis of the correlation between different pairs of AKT and MEK inhibitors

(A) Validation of responses for indicated drugs identified in the drug screen. Cells were treated for 72h with indicated compounds. (HFF, and RMS-ZH004_XC, Mean ± range, n=2 biological replicates; SJRHB13758_X1C, SJRHB13758_X2C, RD, SJRHB010463_X16C, and SJRHB013759_X1C, Mean ± sd, n=3 biological replicates).

(B) Drug response curves for indicated drugs generated with IC-pPDX-35 and SJRHB13758_X2C cells cultured in 2D and 3D conditions. (AZD3463 treatment of SJRHB13758_X2C, n=1 biological replicate, all other SJRHB13758_X2C treatments and torin-2, afuresertib and volasertib treatments of IC-pPDX-35_XC, mean ± range; n=2 biological replicates, all other IC-pPDX-35_XC treatments, mean ± sd; n=3 biological replicates).

(C) Correlation plots and corresponding Pearson correlation coefficients (r) between indicated anti-AKT (upper panels) and anti-MEK (lower panels) drug pairs as calculated from the viability data shown in Figure 4A. (Mean; Pearson correlation).

(D) Cell viability of indicated PPCs treated for 72h with increasing concentrations of the mTOR inhibitor torin-2. (Mean± range; n=2 biological replicates).

Source data are provided as source data file.

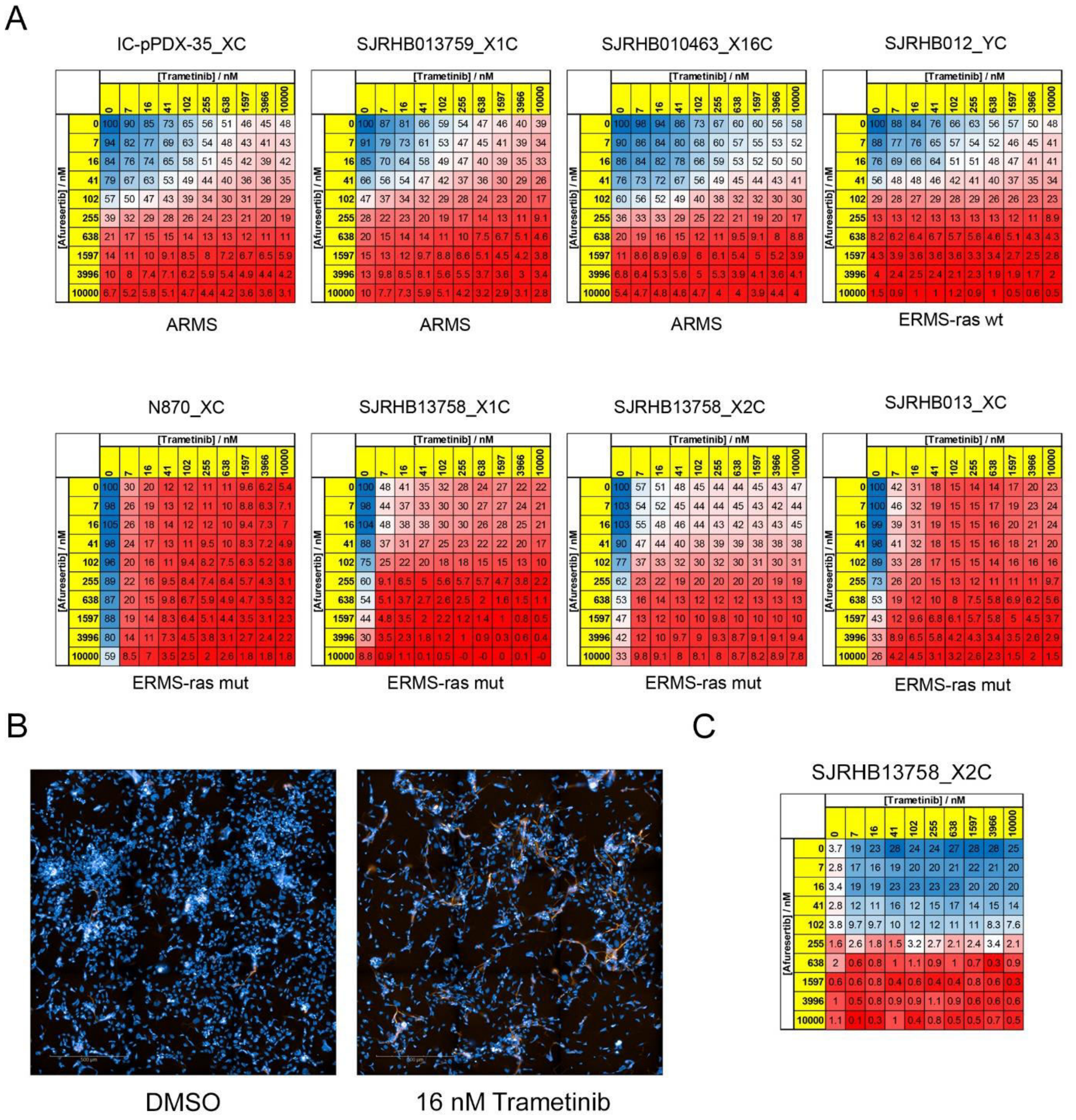

Supplementary Figure 10. Combination of AKT and MEK inhibitors

(A) Percentage of cell viability of indicated PPCs after treatment with a combination matrix of afuresertib and trametinib for three days. (Mean; n=2 biological replicates).

(B) Representative immunofluorescence staining of Myosin Heavy Chain in SJRHB13758_X2C cells treated with vehicle or 16 nM trametinib for three days. Scale bar 500 μm. Displayed images are representative of n=3 biological replicates.

(C) Percentage of Myosin Heavy Chain (MHC) positive SJRHB13758_X2C cells after treatment with the indicated matrix of afuresertib and trametinib for three days. MHC positivity was determined by high throughput immunofluorescence microscopy of cells stained with an anti-MHC antibody. (Mean; n=3 biological replicates).

Source data are provided as source data file.

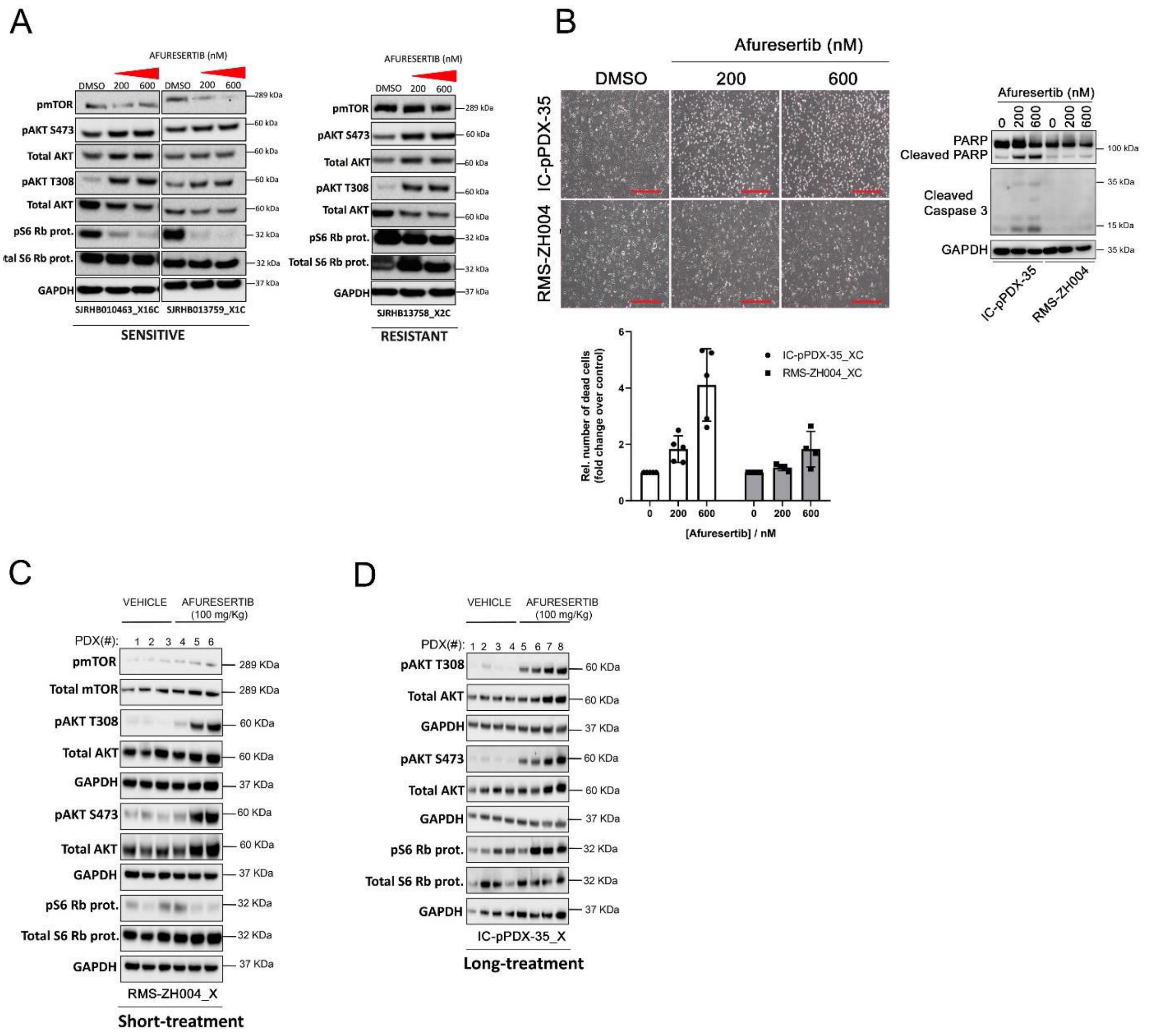

<u>Supplementary Figure 11.</u> Analysis of the effect of afuresertib on the AKT pathway in PPCs and PDXs

(A) Western blot analysis showing expression and phosporylation status of indicated proteins from PPCs treated for 2.5h with afuresertib at 200nM and 600nM or DMSO as control. Both an afuresertib-sensitive (IC-pPDX-35) and -resistant (RMS-ZH004_XC) case is presented. GAPDH was used as loading control. Displayed blots are representative of n=2 independent experiments.

(B, upper left panel) Bright field images of sensitive (IC-pPDX-35) and resistant (RMS-ZH004_XC) PPCs treated for 72h with afuresertib at 200nM and 600nM. Scale bar 200 μm. (B, lower left panel) Quantification of dead cells after treatment with for 72h with afuresertib at 200nM and 600nM by trypan blue assay. (Mean $\pm$ sd; IC-pPDX-35, 5 biological replicates, RMS-ZH004, n=4 biological replicates) (B, right panel) Western blot analysis showing PARP, cleaved PARP and cleaved CASPASE 3 protein levels using extracts from indicated cells. GAPDH was used as loading control. Displayed blots are representative of n=2 biological replicates.

(C and D) Western blot analysis using extracts from tumors treated in vivo for 7 days (C) and 6 weeks (D) with afuresertib (100mg/kg). Displayed blots are representative of n=1 independent experiment.

Source data are provided as source data file.

Supplementary tables.

Supplementary table 1: Genomic regions with focal amplifications

Sample	Target	Gene	Coverage Mean
IC-pPDX29 Cells	chr2:16082070-16087128	MYCN	1298.93
IC-pPDX29 PDX	chr2:16082070-16087128	MYCN	2618.95
	chr2:16080020-16081845	MYCNOS	1256.87
SJRHB011_X PDX	chr8:72755880-72756731	LOC100132891,MSC	2978.68
	chr8:128748315-128753678	MYC	1670.01
	chr8:55528627-55543394	RP1	1153.35
SJRHB011_X Cells	chr8:72755880-72756731	LOC100132891,MSC	3054.45
	chr8:128748315-128753678	MYC	1791.65
	chr8:55528627-55543394	RP1	1068.93
SJRHB012_X Cells	chr12:70002347-70004942	LRRC10	1700.45
SJRHB012_Y PDX	chr12:70002347-70004942	LRRC10	1657.82
	chr12:12508344-12510001	LOH12CR2	1570.38
	chr22:22599200-22599926	VPREB1	1525.2
	chr22:21797048-21805748	HIC2	1291.22
	chr22:22311404-22337147	TOP3B	1248.61
	chr22:21383008-21386847	SLC7A4	1138.45
SJRHB012_Y Cells	chr12:12508344-12510001	LOH12CR2	2372.6
	chr12:70002347-70004942	LRRC10	2250.88
	chr22:22599200-22599926	VPREB1	1460.01
	chr22:21383008-21386847	SLC7A4	1407.42
	chr22:22311404-22337147	TOP3B	1403.83
	chr22:21797048-21805748	HIC2	1301.66
	chr22:21987086-21991615	CCDC116	1246.17
	chr12:69644909-69656342	CPSF6	1178.96
	chr22:21396681-21398538	P2RX6P	1171.17
	chr22:21982378-21984340	YDJC	1151.18
	chr22:21400249-21414866	LOC400891	1109.91
	chr22:22057659-22090071	YPEL1	1084.41
	chr22:21828886-21846383	PI4KAP2	1084.08
	chr12:69979208-69995357	CCT2	1043.04
SJRHB012_Z PDX	chr12:12508344-12510001	LOH12CR2	2408.82
	chr12:70002347-70004942	LRRC10	2406.4
	chr22:22599200-22599926	VPREB1	1689.82
	chr22:22311404-22337147	TOP3B	1424.61

	chr22:21797048-21805748	HIC2	1405.35
	chr22:21383008-21386847	SLC7A4	1389.77
	chr22:21396681-21398538	P2RX6P	1306.97
	chr22:21987086-21991615	CCDC116	1262.96
	chr12:69644909-69656342	CPSF6	1246.96
	chr22:21982378-21984340	YDJC	1160.72
	chr22:22057659-22090071	YPEL1	1146.94
	chr12:69979208-69995357	CCT2	1109.41
	chr22:21400249-21414866	LOC400891	1089.3
	chr22:21400249-21414866	LOC400891	1089.3
	chr22:21828886-21846383	PI4KAP2	1076.63
	chr22:22020273-22049359	PPIL2	1010.26
SJRHB012_Z Cells	chr12:70002347-70004942	LRRC10	1417.62
	chr22:22599200-22599926	VPREB1	1207.43
	chr22:22311404-22337147	TOP3B	1095.01
	chr12:12508344-12510001	LOH12CR2	1087.21
	chr22:21797048-21805748	HIC2	1032.76
	chr22:21383008-21386847	SLC7A4	1023.1
SJRHB013759_X1 Cells	No hits	No hits	No hits
SJRHB013759_X1 PDX	No hits	No hits	No hits
SJRHB010463_X16 Cells	No hits	No hits	No hits
SJRHB010463_X16 PDX	No hits	No hits	No hits
SJRHB010468_X1 Cells	No hits	No hits	No hits
SJRHB010468_X1 PDX	No hits	No hits	No hits
IC-pPDX35 Cells	No hits	No hits	No hits
IC-pPDX35 PDX	No hits	No hits	No hits
N870 Cells	No hits	No hits	No hits
N870 PDX	No hits	No hits	No hits
SJRHB013757_X2 PDX	No hits	No hits	No hits
SJRHB013757_X2 Cells	No hits	No hits	No hits
SJRHB13758_X1 Cells	No hits	No hits	No hits
SJRHB13758_X1 PDX	No hits	No hits	No hits
SJRHB13758_X2 Cells	No hits	No hits	No hits
SJRHB13758_X2 PDX	No hits	No hits	No hits
SJRHB013_X Cells	No hits	No hits	No hits
SJRHB013_X PDX	No hits	No hits	No hits
RMSZH004 Cells	No hits	No hits	No hits
RMSZH004 PDX	No hits	No hits	No hits
RMSZH003 PDX	No hits	No hits	No hits
RMSZH003 Cells	No hits	No hits	No hits

Supplementary table 2: Complete list of drugs used for screenings

Catalog No.	Drug Name
Selleckchem S1004	Veliparib (ABT-888)
Selleckchem S1005	Axitinib
Selleckchem S1006	Saracatinib (AZD0530)
Selleckchem S1007	FG-4592
Selleckchem S1011	Afatinib (BIBW2992)
Selleckchem S1013	Bortezomib (PS-341)
Selleckchem S1014	Bosutinib (SKI-606)
Selleckchem S1018	Dovitinib (TKI-258, CHIR-258)
Selleckchem S1021	Dasatinib
Selleckchem S1023	Erlotinib HCl (OSI-744)
Selleckchem S1025	Gefitinib (ZD1839)
Selleckchem S1028	Lapatinib (GW-572016) Ditosylate
Selleckchem S1029	Lenalidomide (CC-5013)
Selleckchem S1033	Nilotinib (AMN-107)
Selleckchem S1035	Pazopanib HCl
Selleckchem S1039	Rapamycin (Sirolimus)
Selleckchem S1040	Sorafenib Tosylate
Selleckchem S1042	Sunitinib Malate
Selleckchem S1046	Vandetanib (ZD6474)
Selleckchem S1047	Vorinostat (SAHA, MK0683)
Selleckchem S1048	VX-680 (Tozasertib, MK-0457)
Selleckchem S1049	Y-27632 2HCl
Selleckchem S1052	Elesclomol (STA-4783)
Selleckchem S1053	Entinostat (MS-275)
Selleckchem S1055	Enzastaurin (LY317615)
Selleckchem S1060	Olaparib (AZD2281, Ku-0059436)
Selleckchem S1065	GDC-0941
Selleckchem S1067	SB431542
Selleckchem S1068	Crizotinib (PF-02341066)
Selleckchem S1069	AUY922 (NVP-AUY922)
Selleckchem S1070	PHA-665752
Selleckchem S1075	SB216763
Selleckchem S1078	MK-2206 2HCl
Selleckchem S1082	Vismodegib (GDC-0449)
Selleckchem S1092	KU-55933 (ATM Kinase Inhibitor)
Selleckchem S1093	GSK1904529A
Selleckchem S1100	MLN8054
Selleckchem S1107	Danusertib (PHA-739358)
Selleckchem S1113	GSK690693
Selleckchem S1114	JNJ-38877605
Selleckchem S1116	Palbociclib (PD-0332991) HCl
Selleckchem S1119	Cabozantinib (XL184, BMS-907351)
Selleckchem S1120	Everolimus (RAD001)

Selleckchem S1124	BMS-754807
Selleckchem S1130	YM155 (Sepantronium Bromide)
Selleckchem S1133	Alisertib (MLN8237)
Selleckchem S1134	AT9283
Selleckchem S1147	Barasertib (AZD1152-HQPA)
Selleckchem S1153	Roscovitine (Seliciclib,CYC202)
Selleckchem S1164	Lenvatinib (E7080)
Selleckchem S1168	Valproic acid sodium salt (Sodium valproate)
Selleckchem S1171	CYC116
Selleckchem S1180	XAV-939
Selleckchem S1193	Thalidomide
Selleckchem S1200	Decitabine
Selleckchem S1205	PIK-75
Selleckchem S1233	2-Methoxyestradiol (2-MeOE2)
Selleckchem S1267	Vemurafenib (PLX4032, RG7204)
Selleckchem S1362	Rigosertib (ON-01910)
Selleckchem S1378	Ruxolitinib (INCB018424)
Selleckchem S1396	Resveratrol
Selleckchem S1452	Ispinesib (SB-715992)
Selleckchem S1486	AEE788 (NVP-AEE788)
Selleckchem S1487	PHA-793887
Selleckchem S1490	Ponatinib (AP24534)
Selleckchem S1524	AT7519
Selleckchem S1525	MK-1775
Selleckchem S1526	Quizartinib (AC220)
Selleckchem S1532	AZD7762
Selleckchem S1533	R406 (free base)
Selleckchem S1534	Org 27569
Selleckchem S1541	EX 527 (Selisistat)
Selleckchem S1567	Pomalidomide
Selleckchem S1570	KU-60019
Selleckchem S1574	BIRB 796 (Doramapimod)
Selleckchem S1575	RO4929097
Selleckchem S1577	Tie2 kinase inhibitor
Selleckchem S1782	Azacitidine
Selleckchem S1802	Acadesine
Selleckchem S1971	Nicorandil
Selleckchem S2013	PF-573228
Selleckchem S2061	Lovastatin
Selleckchem S2151	LDE225 (NVP-LDE225,Erismodegib)
Selleckchem S2163	PF-4708671
Selleckchem S2180	MLN2238
Selleckchem S2181	MLN9708
Selleckchem S2198	SGI-1776 free base
Selleckchem S2214	AZ 960
Selleckchem S2221	Apatinib
Selleckchem S2235	Volasertib (BI 6727)
Selleckchem S2243	Degrasyn (WP1130)

Selleckchem S2247	BKM120 (NVP-BKM120, Buparlisib)
Selleckchem S2475	Imatinib (STI571)
Selleckchem S2606	Mifepristone
Selleckchem S2626	LY2603618
Selleckchem S2638	NU7441 (KU-57788)
Selleckchem S2660	MK-0752
Selleckchem S2673	Trametinib (GSK1120212)
Selleckchem S2680	Ibrutinib (PCI-32765)
Selleckchem S2686	NVP-BSK805 2HCl
Selleckchem S2696	GDC-0980 (RG7422)
Selleckchem S2697	A-769662
Selleckchem S2719	AMG-900
Selleckchem S2730	Crenolanib (CP-868596)
Selleckchem S2731	AZ 3146
Selleckchem S2742	PHA-767491
Selleckchem S2759	CUDC-907
Selleckchem S2761	NVP-BVU972
Selleckchem S2773	SB705498
Selleckchem S2789	Tofacitinib (CP-690550,Tasocitinib)
Selleckchem S2807	Dabrafenib (GSK2118436)
Selleckchem S2808	GDC-0068
Selleckchem S2817	Torin 2
Selleckchem S2820	TAE226 (NVP-TAE226)
Selleckchem S2824	TPCA-1
Selleckchem S2853	Carfilzomib (PR-171)
Selleckchem S2871	T0070907
Selleckchem S2912	WZ811
Selleckchem S2919	IOX2
Selleckchem S2925	Evacetrapib (LY2484595)
Selleckchem S3012	Pazopanib
Selleckchem S3021	Rimonabant
Selleckchem S4001	Cabozantinib malate (XL184)
Selleckchem S4054	Spironolactone
Selleckchem S4901	JNK-IN-8
Selleckchem S4902	QNZ (EVP4593)
Selleckchem S5001	Tofacitinib (CP-690550) Citrate
Selleckchem S7010	GDC-0152
Selleckchem S7040	AZD3514
Selleckchem S7050	AZ20
Selleckchem S7061	GSK126
Selleckchem S7062	EPZ5676
Selleckchem S7070	GSK J4 HCl
Selleckchem S7083	LDK378
Selleckchem S7085	IWP-2
Selleckchem S7087	GSK2334470
Selleckchem S7094	PF-3758309
Selleckchem S7097	HSP990 (NVP-HSP990)
Selleckchem S7106	AZD3463

Selleckchem S7128	EPZ-6438
Selleckchem S7129	PYR-41
Selleckchem S7130	PR-619
Selleckchem S7132	P5091 (P005091)
Selleckchem S7138	BMS-833923
Selleckchem S7145	AZD1080
Selleckchem S7152	C646
Selleckchem S7153	10058-F4
Selleckchem S7173	AVL-292
Selleckchem S7234	IOX1
Selleckchem S7237	OG-L002
Selleckchem S7256	SGC-CBP30
Selleckchem S7257	CNX-774
Selleckchem S7265	MM-102
Selleckchem S7281	JIB-04
Selleckchem S7294	PFI-2
Selleckchem S7304	CPI-203
Selleckchem S7307	GSK2606414
Selleckchem S7330	6H05
Selleckchem S7332	K-Ras(G12C) inhibitor 9
Selleckchem S7337	SH-4-54
Selleckchem S7360	OTX015
Selleckchem S7440	LEE011
Selleckchem S7461	LDC000067
Selleckchem S7462	PI-1840
Selleckchem S7508	JNK Inhibitor IX
Selleckchem S7519	GNF-5837
Selleckchem S7521	Afuresertib (GSK2110183)
Selleckchem S7554	GDC-0994
Selleckchem S7570	UNC0379
Selleckchem S7574	GSK-LSD1 2HCl
Selleckchem S7581	GSK J1
Selleckchem S7587	INCB024360
Selleckchem S7591	BRD4770
Selleckchem S7597	BV-6
Selleckchem S7611	EI1
Selleckchem S7618	MI-2 (Menin-MLL Inhibitor)
Selleckchem S7638	LDC1267
Selleckchem S7656	CPI-360
Selleckchem S7665	CH5183284 (Debio-1347)
Selleckchem S7679	YK-4-279
Selleckchem S7693	AZD6738
Selleckchem S7707	Verdinexor (KPT-335)
Selleckchem S7748	EPZ015666
Selleckchem S7799	Pexmetinib (ARRY-614)
Selleckchem S7818	Pexidartinib (PLX3397)
Selleckchem S7843	BI-847325
Selleckchem S7906	PFI-4

Selleckchem S7910	Epacadostat (INCB024360)
Selleckchem S8031	NSC 23766
Selleckchem S8044	BMS-345541
Selleckchem S8057	Pacritinib (SB1518)
Selleckchem S1001	ABT-263
Selleckchem S7163	Dyngo 4a
Selleckchem S1057	Obatoclax
Selleckchem S7015	Birinapant
Selleckchem S1208	Doxorubicin
Selleckchem S1225	Etoposide
Selleckchem S1241	Vincristine
Selleckchem S8041	GDC-0973
Selleckchem S7205	Idasanutlin
ApexBio A1910	JQ-1
Selleckchem S2780	iBET
Sigma-Aldrich D7693	Dynasore
Sigma-Aldrich H7779	Fenretinide
