# Supplementary material for: Phenotypic profiling with a living biobank of primary rhabdomyosarcoma unravels disease heterogeneity and AKT sensitivity
Source: Nat Commun. 2020 Sep 15;11:4629. doi: 10.1038/s41467-020-18388-7 (PMC7492191; doi:10.1038/s41467-020-18388-7)
Supplement: Supplementary file 3 — Reporting Summary [file 41467_2020_18388_MOESM3_ESM.pdf]

natureresearch

Corresponding author(s): Beat W. Schäfer

Last updated by author(s): Jul 2, 2020

Reporting Summary

Nature Research wishes to improve the reproducibility of the work that we publish. This form provides structure for consistency and transparency in reporting. For further information on Nature Research policies, see Authors & Referees and the Editorial Policy Checklist .

Statistics

For all statistical analyses, confirm that the following items are present in the figure legend, table legend, main text, or Methods section.

n/a	Confirmed	
☐	☒	The exact sample size (*n*) for each experimental group/condition, given as a discrete number and unit of measurement
☐	☒	A statement on whether measurements were taken from distinct samples or whether the same sample was measured repeatedly
☐	☒	The statistical test(s) used AND whether they are one- or two-sided<br>*Only common tests should be described solely by name; describe more complex techniques in the Methods section.*
☐	☒	A description of all covariates tested
☐	☒	A description of any assumptions or corrections, such as tests of normality and adjustment for multiple comparisons
☐	☒	A full description of the statistical parameters including central tendency (e.g. means) or other basic estimates (e.g. regression coefficient) AND variation (e.g. standard deviation) or associated estimates of uncertainty (e.g. confidence intervals)
☐	☒	For null hypothesis testing, the test statistic (e.g. *F*, *t*, *r*) with confidence intervals, effect sizes, degrees of freedom and *P* value noted<br>*Give P values as exact values whenever suitable.*
☒	☐	For Bayesian analysis, information on the choice of priors and Markov chain Monte Carlo settings
☒	☐	For hierarchical and complex designs, identification of the appropriate level for tests and full reporting of outcomes
☒	☐	Estimates of effect sizes (e.g. Cohen's *d*, Pearson's *r*), indicating how they were calculated

Our web collection on statistics for biologists contains articles on many of the points above.

Software and code

Policy information about availability of computer code

Data collection	BioRad Image Lab 5.2.1 was used for chemiluninescence detection of Western Blots.<br>Zeiss Zen 2012 (blue edition) 1.1.2.0 was used for epifluorescence microscopy.<br>Harmony 4.9 was used for automated microscopy with an Perkin Elmer Operetta high content imaging system<br>BioTek Gen5 2.07.17 was used for measurement of colorimetric assays (WST).
Data analysis	Prism 8.0.0 was used for all statistical analyses.<br>Chromosome Analysis Suite 3.2.0.1252 was used for analysis of array CGH data.<br>Whole-Exome sequencing Pipeline web tool (WEP) (https://bioinformatics.cineca.it/wep/index.php) was used for analysis of exom seq data.<br>Synergy Finder webtool (https://synergyfinder.fimm.fi/synergy/20200618090506857638/) was used for calculation of drug synergism.<br>dChip 2011 was used for unsupervised hierarchical clustering analysis.

For manuscripts utilizing custom algorithms or software that are central to the research but not yet described in published literature, software must be made available to editors/reviewers. We strongly encourage code deposition in a community repository (e.g. GitHub). See the Nature Research guidelines for submitting code & software for further information.

Data

Policy information about availability of data

All manuscripts must include a data availability statement. This statement should provide the following information, where applicable:

- Accession codes, unique identifiers, or web links for publicly available datasets
- A list of figures that have associated raw data
- A description of any restrictions on data availability

Array CGH (Fig.3A), Exom seq (Fig.3B and D) and DNA methylation (Fig.3C) data is deposited on the dbGAP databse under the accession number phs002051.v1.p1. PPCs can be obtained from the corresponding author through an MTA.

Field-specific reporting

Please select the one below that is the best fit for your research. If you are not sure, read the appropriate sections before making your selection.

[x] Life sciences [ ] Behavioural & social sciences [ ] Ecological, evolutionary & environmental sciences

For a reference copy of the document with all sections, see nature.com/documents/nr-reporting-summary-flat.pdf

Life sciences study design

All studies must disclose on these points even when the disclosure is negative.

Sample size	Sample size was selected based on expected effect size and variability between treatment groups.
Data exclusions	No data was excluded
Replication	All experiments were performed at least twice, in most cases more than this. All replicates were succesful and are included.
Randomization	All mice were randomly assigned to individual treatment groups at the beginning of treatment.
Blinding	The in vivo experiment was not blinded.

Reporting for specific materials, systems and methods

We require information from authors about some types of materials, experimental systems and methods used in many studies. Here, indicate whether each material, system or method listed is relevant to your study. If you are not sure if a list item applies to your research, read the appropriate section before selecting a response.

Materials & experimental systems

n/a	Involved in the study	
[ ]	[x]	Antibodies
[ ]	[x]	Eukaryotic cell lines
[x]	[ ]	Palaeontology
[ ]	[x]	Animals and other organisms
[ ]	[x]	Human research participants
[x]	[ ]	Clinical data

Methods

n/a	Involved in the study	
[x]	[ ]	ChIP-seq
[x]	[ ]	Flow cytometry
[x]	[ ]	MRI-based neuroimaging

Antibodies

Antibodies used

The following antibodies were used:

For Immunohistochemsitry:
Desmin (Novocastra Laboratories Ltd, NCL-L-DES-DERII) (1:200)
Myogenin (Cell Marque Lifescreen, 296M-14) (1:100)
AP2b (Santa Cruz,H-87) (1:400)
Cleaved Caspase-3 (Cell Signaling Technology, #9661) (1:500)
Glut-1 (Cell Marque Lifescreen Ltd., CMC35511020) (1:300)

For Western Blot:
anti-phospho-mTOR (CellSignaling #2971S),
anti-mTOR (CellSignaling #2983S),

anti-phospho-S6 ribosomal protein (CellSignaling #2211S),
anti-S6 ribosomal protein (CellSignaling #2217S),
anti-phospho-AKT Thr308 (CellSignaling #9275S),
anti-phospho-Akt Ser473 (CellSignaling #9271S),
anti-AKT (CellSignaling #9272),
anti-GAPDH (CellSignaling #2118S),
anti-PARP (CellSignaling #9542S),
anti-cleaved CASPASE 3 (CellSignaling #9664P).
anti-P53 (DO-1; ThermoFisher Scientific #AHO0152)
anti-FOXO1 (H128; Santa Cruz Biotechnology sc-11350)
anti-mouse IgG HRP-labeled (CellSignaling #7076S)
anti-rabbit IgG HRP-labeled (CellSignaling #7074S)

For Immunofluorescence:
anti-MHC antibody (MF-20; Developmental Studies Hybridoma Bank)
Donkey anti-mouse IgG Alexa-594 labeled (Thermofisher Scientific, A11032)

Validation

Antibodies used for Immunohistochemsitry:
Desmin (Novocastra Laboratories Ltd, NCL-L-DES-DERII): Validation data for IHC is available on the LEICA homepage
Myogenin (Cell Marque Lifescreen, 296M-14): Validation for IHC staining is available on the homepage of the manufacturer
AP2b (Santa Cruz,H-87): Validation data is available on the homepage of the manufacterer. This antibody was also validated by the group of the authors for IHC (Wachtel et al, JCO 2006).
Cleaved Caspase-3 (Cell Signaling Technology, #9661): Validation data for IHC is available on the homepage of the manufacterer
Glut-1 (Cell Marque Lifescreen Ltd., CMC35511020): Validation data for IHC is available on the homepage of the manufacterer

Antibodies used for Western Blots
Validation data for the application Western Blot for all the following antibodies from CellSignaling is available on the CellSignaling homepage:
anti-phospho-mTOR (CellSignaling #2971S)
anti-mTOR (CellSignaling #2983S)
anti-phospho-S6 ribosomal protein (CellSignaling #2211S),
anti-S6 ribosomal protein (CellSignaling #2217S),
anti-phospho-AKT Thr308 (CellSignaling #9275S),
anti-phospho-Akt Ser473 (CellSignaling #9271S),
anti-AKT (CellSignaling #9272),
anti-GAPDH (CellSignaling #2118S),
anti-PARP (CellSignaling #9542S),
anti-cleaved CASPASE 3 (CellSignaling #9664P).

anti-P53 (DO-1; ThermoFisher Scientific #AHO0152) was validated by for Western blot comparing p53-positive and ko cells by the manufacturer as depicted on their homepage.
anti-FOXO1 (H-128; Santa Cruz Biotechnology sc-11350) was used in numerous publications for Western Blot listed on the SantaCruz website.

Antibodies used for immunofluorescence
anti-MHC (MF-20; Developmental Studies Hybridoma bank) was used in numerous publications for immunofluorescence listed on the Developmental Studies Hybridoma Bank website.

Eukaryotic cell lines

Policy information about cell lines

Cell line source(s)

The alveolar rhabdomyosarcoma cell lines RH4 and Rh30 and the embryonal rhabdomyosarcoma cell line Rh36 were all provided by Peter Houghton, Greehey Children's Cancer Research Institute, San Antonio, Texas, USA. The embryonal rhabdomyosarcoma cell line RD was purchased from ATCC.

Authentication

All cell lines were authenticated by short tandem repeat analysis (STR profiling) and positively matched with reference data

Mycoplasma contamination

Cell lines were regularly tested for Mycoplasma and were Mycoplasma free

Commonly misidentified lines
(See ICLAC register)

None as far as we know.

Animals and other organisms

Policy information about studies involving animals; ARRIVE guidelines recommended for reporting animal research

Laboratory animals	NOD scid gamma (NSG) mice were used for all animal experiments.<br>Animals were bred in-house and health screens (quarterly) were conducted in accordance with FELASA guidelines for health monitoring of rodent colonies, to confirm their free statues of known pathogens in accordance with FELASA screens. No clinical signs were detected. Animals were housed in groups of 4-6 mice per individually ventilated cage in a 12 h light dark cycle (06:30-18:30 light; 18:30-06:30 dark), with controlled room temperature (21 ± 1 °C) and relative humidity (40-60 %). The cages contained 1-1.5 cm layer of animal bedding, and with environmental enrichment including cardboard Box-tunnel and crinkled paper nesting material. Animals had access to food and water ad libitum.
Wild animals	No wild animals were used.
Field-collected samples	This study did not involve samples collected from the field
Ethics oversight	Animal studies were performed according to the FELASA guidelines

Note that full information on the approval of the study protocol must also be provided in the manuscript.

Human research participants

Policy information about studies involving human research participants

Population characteristics	One 10 year old, female patient with an alveolar rhabdomyosarcoma.
Recruitment	The patient presented at the University Children`s Hospital during the course of the study and was then asked for consent to perform the drug profiling experiments on the tumor cells.
Ethics oversight	The usage of the patient material was approved by the local ethics committee (REQ-2016-00108).

Note that full information on the approval of the study protocol must also be provided in the manuscript.
